# CBRNe Personal Protective Equipment Is Not a Hindrance to Lifesaving Procedures in Prehospital Settings: A Prospective, Repeated-Measures Observational Study

**DOI:** 10.3390/epidemiologia6040057

**Published:** 2025-09-23

**Authors:** Stefano Innocenzi, Fabio Ingravalle, Massimo Maurici, Daniela Di Rienzo, Danilo Casciani, Michelangelo Cesare Rinella, Antonio Vinci, Eliana Giuffré, Nicoletta Trani, Stefania Iannazzo, Narciso Mostarda

**Affiliations:** 1 Azienda Regionale Emergenza Sanitaria ARES 118, Via Portuense 240, 00149 Roma, Italy; sinnocenzi@ares118.it (S.I.);; 2 Department of Biomedicine and Prevention, Tor Vergata University of Rome, Viale Montpellier 1, 00167 Roma, Italy; 3 Local Health Authority “ASL Roma 1”, Borgo Santo Spirito 3, 00193 Roma, Italy; 4 Department of Public Health and Infectious Diseases, Sapienza University of Rome, 00185 Roma, Italy

**Keywords:** frontline workers, Emergency Medical Services, accidents, airway management, nursing, personal protective equipment

## Abstract

**Objectives**: The primary objective was to compare the usage of Hazardous Materials (HazMat) Protective Personal Equipment (PPE) and ordinary PPE when performing basic and advanced health care support maneuvers in a prehospital setting, evaluating the effectiveness of several procedures, defined as the mean success rate of each. The secondary objective was to evaluate the presence of a learning effect, with improvements in the success rate and/or procedure timing. **Methods**: This was a prospective within-subjects (repeated-measures) study conducted on Emergency Medical Services (EMS) responders within their Chemical-Biological-Radiological-Nuclear-Explosive (CBRNe) training institutional programme. Volunteers performed a trial sequence of eight lifesaving procedures four times. During the first trial sequence, they wore standard clothing; during the three successive trials, they wore full HazMat PPE equipment. The primary outcomes were changes in success rate and time interval across the four trials. **Results**: A total of 146 EMS responders volunteered for the experiment. Procedure success rates remained high overall, with the most notable initial drop observed for video-assisted intubation (≈−10%). The only statistically significant delay in the first HazMat trial compared with baseline was for intravenous access (median +30 s; *p* < 0.001). In the two successive HazMat trials, success rates and timings improved, with median values coming close to baseline. However, only 61% of participants completed the entire drill due to tolerance limits of the equipment. **Conclusions**: HazMat PPE, while physically and ergonomically demanding, has minimal impact on most lifesaving procedures, though it may reduce intubation success and delay intravenous access. Tolerance to prolonged use is a key limitation, but dexterity improves rapidly with brief practice. EMS responders can benefit from continuous training practice, while manufacturers could explore ergonomic and tolerance improvements in their PPE equipment.

## 1. Introduction

### 1.1. Background and Rationale

Chemical-Biological-Radiological-Nuclear-Explosive (CBRNe) accidents are rare and challenging scenarios, posing extreme difficulties to Emergency Medical Service (EMS) providers [1]. EMS responders often need to act in conjunction with other institutional figures, such as firefighters, police, or civil defense organizations, in situations whose incident command system may vary depending on the involved organization and the level of threat [2].

The effectiveness of incident command is essential for the successful management of such events, as correct decisions must quickly be made in regards to patient care and personnel safety, with the possibility of being caught in ethically difficult circumstances [3]. Any incident commander must therefore be capable of evaluating both the context situation, the patients’ clinical needs, and the personnel’s actual or potential exposure to any professional risk [4,5].

Currently, the staple of all safety measures for personnel involved in CBRNe accidents consists of the correct use of personal protective equipment (PPE), which in such scenarios is different from ordinary equipment, as it also gives protection against Hazardous Materials (HazMat) [6]. Such PPE can be used by first responders and health care personnel alike. However, evidence of its effectiveness when performing lifesaving procedures is mixed. Some authors have argued that procedures are less comfortable and more stressful under HazMat PPE when compared with ordinary PPE, and others that procedures are more cumbersome and take a longer time [7,8].

On the other hand, other studies evidenced the feasibility of HazMat PPE in several lifesaving procedures and did not find significant differences in intubation time and efficiency [6,9,10]. Most of the literature on this subject is focused on the hospital setting, with little to no evidence of HazMat usage by EMS responders in prehospital scenarios.

### 1.2. The EMS Response in the Lazio Region

The Italian National Health System (NHS) is a Beveridge system, with public funding and universal and free coverage. By law, the NHS is the only authority tasked with responding to medical rescue and medical health emergency calls in territorial (i.e., non-hospital) settings in Italy [11]. In the Lazio region, which is a very populous Italian region with over 4 million inhabitants and home to the capital city of Rome, this task is assigned to the Agenzia Regionale Emergenza Sanitaria 118 (ARES—Regional Health Emergency Agency), which is a component of the Italian NHS. ARES 118 is also embedded in the regional epidemiological surveillance system, as the EMS in Italy also has a monitoring role during pandemics [12].

ARES 118 operates with a fleet of over 200 EMS vehicles (mostly ambulances and some cars) and three helicopters; every vehicle, by regional law, is staffed with at least one health professional (registered nurse or medical doctor). For most vehicles, the staff also includes a driver and a support operator (stretcher bearer). All personnel are trained in Basic Life Support with Defibrillator (BLS-D), and health professionals are also trained in Advanced Life Support (ALS) as a requirement for being operational. The response offer is enhanced by a HazMat team and an Urban Search-and-Rescue team, both drafted on demand from active operating personnel in case of a hazardous event, and in some cases deployed in international rescue operations.

### 1.3. Study Objectives

The primary objective of this investigation was to compare the usage of HazMat PPE and ordinary PPE when performing basic and advanced health care support in a training environment in terms of procedure effectiveness. The null hypothesis was that there is no difference in timing and success rate for the most common lifesaving procedures that a HazMat responder team is expected to perform compared with ordinary PPE usage. The secondary objective was to evaluate the presence of a learning effect, with improvements in the success rate and/or procedure timing.

## 2. Materials and Methods

### 2.1. Design and Setting

This is a prospective within-subjects (repeated-measures) observational study conducted on EMS responders within their CBRNe training institutional programme. No patient or human subject was involved in the study. The study was not registered on any repository; registration was waived, since, per the International Committee of Journal Editors (ICMJE) guidelines, it was not mandatory because the study did not consist of a clinical trial (its purpose was to evaluate only the practitioners) [13].

The study was conducted from 11 July 2022 to 30 October 2023 on a large convenience sample consisting of the entirety of EMS HazMat personnel of ARES 118. Course participation is a strict requirement for HazMat team membership and, therefore, eligibility.

**Figure 1:**
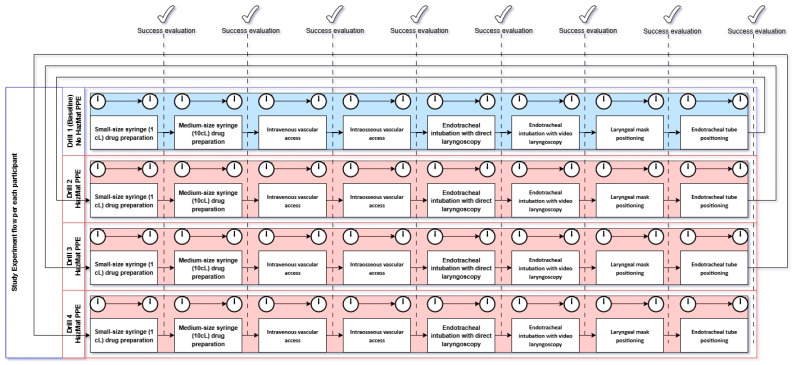
Study flow chart. PPE: Personal Protective Equipment.

All study participants were EMS responders employed by ARES 118 during 2022 and 2023, each having received basic EMS training and specific training to perform the procedures investigated in an ordinary setting. They attended the internal CBRNe training course organized by the agency to enlist in the HazMat team; participation was voluntary and occurred without institutional scheduling or selection. Being either registered nurses or medical doctors, they already had certified training in both BLS-D and ALS. Age, gender, and professional experience (in years) of each professional were recorded. Each participant performed a drill series of lifesaving procedures on a standardized simulation manikin under supervision of a tutor (SI). The manikin used was a standard-use training manikin (Resusci Anne QCPR AW with Airway Head model, Laerdal^®^, Stavanger, Norway). For each procedure, participants were classified as either expert (had already performed the procedure in vivo at least once in their lifetime) or naïve (never performed the procedure in vivo). Each procedure followed the operational protocol derived from ALS guidelines with objective success criteria pre-specified and applied uniformly by the tutor [14]. Two outcome types were recorded for each procedure and participant: technical success (binary), defined as tutor’s judgement or objective signs on the manikin (i.e., pulmonary expansion for ventilation), and procedure interval (in seconds), defined as the interval timed from task start to successful final step, according to Spaite’s time/interval model [15]. The measurements were taken at the ARES118 training facility, where each individual was scheduled to be trained for HazMat equipment usage, contextually during the trials, which constituted the conclusive part of the training course and were performed right after the first lesson. In the case of missing data (e.g. no timing due to failure in performing a procedure), timing failures were replaced with “closest previous timing”. The trial sequence was structured as follows (Figure 1):

1. Baseline
  - Participants wore their standard work clothing and single-glove protection;
  - They performed each of the eight procedures once.
2. HazMat PPE
  - Participants wore full HazMat PPE (Type 3B liquid-tight chemical protective suit, full-face mask with integrated particulate and gas/vapor filter, butyl rubber gloves, and CBRNe-compliant protective boots);
  - They repeated the full sequence of eight procedures three times each.

This resulted in four successive trials per individual (one baseline trial, one first-time HazMat trial, two successive HazMat trials).

The procedures investigated were performed in fixed order:

1. Small-sized syringe (1 cL) drug preparation;
2. Medium-sized syringe (10 cL) drug preparation;
3. Intravenous vascular access;
4. Intraosseous vascular access;
5. Endotracheal intubation with direct laryngoscopy;
6. Endotracheal intubation with video laryngoscopy;
7. Laryngeal mask positioning;
8. Endotracheal tube positioning.

The study was reported according to the STrengthening the Reporting of Observational studies in Epidemiology (STROBE) statement [16]. Its checklist is available in Supplementary Materials.

### 2.2. Statistical Analysis

This single-blind analysis was carried out at the Department of Biomedicine and Prevention of the University of Rome “Tor Vergata” using STATA v.17.0. (StataCorp^®^, College Station, TX, USA). All analyses were conducted in pseudo-anonymous form, as each participant was identified via their unique ID numeric code, so that their identity was not known by researchers.

For descriptive analysis, qualitative variables were synthesized using frequencies and percentages, while quantitative variables were synthesized using means and standard deviation (SD) or median and interquartile range (IQR). For inferential analysis, Cochran’s Q test was used to find differences in success rates between successive trials for each procedure; post hoc McNemar test (Bonferroni-corrected) was then used for pairwise comparison. The cochranq Stata package was used for such analysis [17]. The Shapiro–Wilk test was used to verify the normality of procedure timing data; since distributions were not normal, non-parametric tests of hypothesis were used for timing comparison. To investigate the differences in procedure timing (Δ) across trials, for each subject and each procedure, three Δ variables were created:

- Δ_12_ = Time_(Trial2)_ − Time_(Trial1)_;
- Δ_23_ = Time_(Trial3)_ − Time_(Trial2)_;
- Δ_34_ = Time_(Trial4)_ − Time_(Trial3)_.

If some trial was not associated with a valid timing due to procedure failure, the closest previous timing was used instead, i.e., if Trial 1 = 10 s, Trial 2 = failure, Trial 3 = 6 s, then Δ_12_ = undefined, Δ_23_ = 4 s. Hodges–Lehmann median difference (and its confidence intervals) were then calculated for each procedure (using the cendif Stata package), and the Wilcoxon rank-sum test was used for statistical significance testing [18]. The level of significance for all inferential analyses was set at *p* = 0.05. It was initially planned to perform a logistic analysis to investigate the role of potential moderators (such as gender, age, or experience) on the chance of delivering an effective procedure; however, due to the high success rates recorded, such an analysis would have been completely uninformative and was therefore omitted.

## 3. Results

A total of 146 EMS responders (74 males, 72 females) were included in the study. Most participants (130) were registered nurses, and only 16 were medical doctors. All participants had field experience in EMS response, as it is their daily professional activity. Their professional experience varied, with a median of 8 years of professional activity (IQR: 3–18). On average, they required 75 min to complete all the trials (SD: 17 min); however, some (61 practitioners, 42%) did not tolerate the HazMat PPE long enough to complete all the procedures. Male practitioners were twice as likely to complete the full drill as females (95% CI: 1.02–3.91, *p* = 0.04), while age and professional experience were not associated with this occurrence. Details on procedure success rates are available in Table 1, as well as the number of participants in each stage and their level of experience; Table 2 contains details on procedure timings. Among the procedures performed, the only statistically significant difference in success rate (10%) was for endotracheal intubation with direct laryngoscopy; Cochran’s Q was significant (*p* = 0.042), but pairwise McNemar tests were not significant after Bonferroni correction. Regarding timing, intravenous vascular access positioning was slower in the first HazMat trial when compared with baseline (≈30 s, *p* < 0.001). Time reduction was obtained with successive trials (≈12 s, *p* < 0.001), suggesting an adaptive and learning effect. All other procedures did not show any difference in timing when performed in a HazMat suit compared with baseline.

## 4. Discussion

Preparedness to CBRNe accidents is key to their effective management and mitigation of their effects, and it involves awareness, training, and knowledge of what is possible and what is acceptable to do [19]. In our study, we found that while wearing HazMat protection, the technical success rate of the most common lifesaving procedures remains high, at levels comparable with the standard of care. The most significant drop in performance (−10%) was recorded for endotracheal intubation with video laryngoscopy, which is, however, notoriously difficult, with real-life reported success rates ranging from 35% to 81% [20]. However, with minimal, successive practice (Trials 3–4), EMS responders rapidly regained much of their baseline proficiency: success rates rebounded toward baseline levels, and median time intervals decreased significantly between Trials 2 and 4. Moreover, the difference was not statistically significant in post hoc pairwise comparison.

Interestingly, and unexpectedly, the differences in duration when comparing baseline with the first HazMat trial for most procedures were mostly trivial and not statistically significant, the only exception being a significant delay (≈30 s) when picking intravenous vascular access (*p* < 0.001). Median times decreased significantly with successive attempts, suggesting that personnel may adapt and improve their dexterity while in the field. However, since it is difficult to acquire first-hand expertise due to the relative rarity of CBRNe events, ensuring proper training in simulated scenarios is a viable option to increase responders’ expertise and autonomy [21,22]. Training in emergency settings is a well-known foundation for efficient responses after all, and is also a fundamental element of preparedness in healthcare systems during pandemic times: moreover, during the COVID-19 pandemic, a decrease in both training frequency and bystander cardiopulmonary resuscitation was observed. [23,24]

It should be noted, however, that the use of HazMat equipment may still be problematic in the case of prolonged exposure. In our study, even if 139 (95%) participants completed seven out of eight tests, only 81 (61%) tolerated the equipment long enough to complete the entire drill. Our mean duration time (75 min) is in line with other studies found in the literature that report similar limitations, with tolerance even as low as 20 min in dry–harsh conditions [25]. Such findings suggest the importance of optimizing equipment design, but also the physical and psychological fatigue responders may feel during extended operations. We found a statistically significant difference (OR ≈ 2.0; CI 1.02–3.91) between male and female completion rates; however, given the ample confidence interval, such a result should be interpreted with caution. Several unmeasured factors may have contributed to this difference, including body size, thermoregulation, and the fit or ergonomics of the PPE itself, which is typically designed around standard anthropometric models that may not equally accommodate all users. Individual characteristics, such as physical endurance and tolerance to heat stress, could also play a role. Therefore, while the observed association is noteworthy, specific studies are needed before drawing conclusions on any gender-related differences in PPE tolerance.

Crucially, responders’ ability to perform efficiently may be further compromised if healthcare systems are under strain. Emergency department overcrowding and high bed occupancy rates in acute hospitals may create a backward cascade effect that may affect prehospital EMS responses by prolonging response intervals and diverting resources, exacerbating phenomena such as ambulance ramping and hospital boarding [26,27,28]. These pressures can translate into longer EMS response times, reduce the availability of frontline personnel and vehicles, and increase logistical complexity. In this context, emerging telemedicine platforms have begun to demonstrate feasibility and safety in the field, in both prehospital and territorial settings, offering real-time remote support that may help EMS responders overcome some of the dexterity and visual limitations imposed by full CBRNe PPE [29,30]. Integrating such platforms into CBRNe training and response protocols may further compress critical time intervals and bolster both responder confidence and patient safety.

### Limitations

This was a single-center study conducted on a cohort of EMS responders that was selected on a voluntary basis to be enlisted in the HazMat team. This may introduce a selection bias toward responders who are more motivated compared with the average EMS responder. However, because HazMat response teams are staffed by design with highly trained and motivated practitioners, the characteristics of our sample are likely to reflect those of operational CBRNe units, supporting the broader applicability of our findings. Moreover, this limitation is purely speculative, as there is no economic/career incentive in joining HazMat teams. Among the study limitations are the usage of manikins, which may not fully replicate patient movements or tissue resistance in nonstandard conditions such as CBRNe environments; of course, nothing can be comparable to actual in vivo practice. However, it would be ethically impossible to design such a trial; therefore, simulation results are the closest-to-reality evidence available. The simulation manikin environment, while standardized, cannot fully reproduce the unpredictability of a contaminated scene or patient movement. Additionally, all participants performed the baseline (standard attire) trial first, potentially impacting successive effects. Future studies should randomize trial order or include a wash-out period. Future research developments could consider multi-center designs, inclusion of novice practitioners, and evaluation of cognitive workload and team-based dynamics under CBRNe conditions—all elements that were not subject to current experimentation.

## 5. Conclusions

HazMat equipment has little to no impact on procedural speed and technical success of most lifesaving procedures. The exceptions were a reduction in intubation success rate and an increase in intravenous vascular access timing. Equipment tolerance remains a major operative limitation, especially in prolonged usage, while dexterity may rapidly improve with limited practice. The implications of these findings involve both EMS provider institutions and PPE manufacturers. Institutions should provide continuous EMS training practice for responders and institutionalize HazMat PPE usage in their curricula if teams are expected to perform their activity in a HazMat environment. Manufacturers could use this evidence to identify areas of improvement in the equipment itself, as the major hindrance identified in the study (and in accordance with the published literature) lies in ergonomics and tolerance of PPE gear.

## Figures and Tables

**Table 1:** Trial success rates and number of EMS responders per trial.

Procedure	T1 (Baseline) Success Rate	T1 (Baseline) Participants (Experts)	T2 Success Rate	T2 Participants (Experts)	T3 Success Rate	T3 Participants (Experts)	T4 Success Rate	T4 Participants (Experts)	Cochran’s Q Test *p*-Value
Small-sized syringe (1 cL) drug preparation	100.0%	146 (146)	99.3%	146 (146)	99.3%	146 (146)	99.3%	146 (146)	*p* ≈ 1.000
Medium-sized syringe (10 cL) drug preparation	100.0%	146 (146)	99.3%	146 (146)	99.3%	146 (146)	98.6%	146 (146)	*p* ≈ 1.000
Intravenous vascular access	99.3%	146 (146)	98.6%	146 (146)	98.6%	146 (146)	99.3%	146 (146)	*p* = 0.677
Intraosseous vascular access	96.6%	146 (108)	96.6%	146 (108)	96.6%	146 (108)	95.9%	146 (108)	*p* = 0.962
Endotracheal intubation with direct laryngoscopy	68.5%	146 (71)	71.2%	146 (71)	76.0%	146 (71)	78.1%	146 (71)	*p* = 0.042 *
Endotracheal intubation with video laryngoscopy	83.4%	145 (71)	73.6%	144 (71)	77.8%	144 (71)	80.1%	144 (71)	*p* = 0.058
Laryngeal mask positioning	100.0%	139 (113)	97.8%	139 (113)	99.3%	139 (113)	99.3%	139 (113)	*p* = 0.058
Endotracheal tube positioning	100.0%	85 (83)	98.8%	85 (83)	98.8%	85 (83)	100.0%	85 (83)	*p* ≈ 1.000

*: Pairwise comparisons not significant after Bonferroni adjustment.

**Table 2:** Median procedure intervals (seconds), pairwise Hodges–Lehmann differences (seconds), and *p*-values.

Procedure	Baseline (Median; IQR)	Δ_12_ (95% CI); *p*-Value	Δ_23_ (95% CI); *p*-Value	Δ_34_ (95% CI); *p*-Value
Small-sized syringe (1 cL) drug preparation	37; 29–45	2; (−1; 5); *p* = 0.178	−5; (−8; −3); *p* < 0.001	−3; (−6; 0); *p* = 0.023
Medium-sized syringe (10 cL) drug preparation	49; 41–59.5	1; (−2; 5); *p* = 0.471	−6; (−9; −2); *p* = 0.002	−3; (−6; 0); *p* = 0.085
Intravenous vascular access	49; 40–59	30; (24; 35); *p* < 0.001	−12; (−18; −5); *p* < 0.001	−4; (−10; 1); *p* = 0.151
Intraosseous vascular access	73; 54–91	0; (−6; 7); *p* = 0.898	−9; (−15; −4); *p* < 0.001	−5; (−10; −1); *p* = 0.028
Endotracheal intubation with direct laryngoscopy	45; 34–61	3; (−3; 8); *p* = 0.322	−7; (−12; −2); *p* = 0.006	−1; (−5; 3); *p* = 0.711
Endotracheal intubation with video laryngoscopy	57; 39.5–78	5; (−3; 14); *p* = 0.225	−14; (−21; −6); *p* < 0.001	−2; (−9; +4); *p* = 0.478
Laryngeal mask positioning	22; 17–30	−1; (−3; 1); *p* = 0.478	−1; (−3; 0); *p* = 0.123	−1; (−3; 0); *p* = 0.066
Endotracheal tube positioning	23; 19–28	2; (−1; 4); *p* = 0.156	−5; (−7; −2); *p* < 0.001	−1; (−3; 0); *p* = 0.123

## Data Availability

The raw data supporting the conclusions of this article will be made available by the authors on request.

## Supplementary Materials

The following supporting information can be downloaded at: https://www.mdpi.com/article/10.3390/epidemiologia6040057/s1, STROBE Checklist.

## Abbreviations

The following abbreviations are used in this manuscript:

CBRNe	Chemical-biological-radiological-nuclear-explosive
EMS	Emergency Medical Service
HazMat	Hazardous Materials
NHS	National Health System
PPE	Personal protective equipment
SD	Standard deviation
